# LATE-LIFE DISABILITY FOLLOWING THE ACTION FOR HEALTH IN DIABETES (LOOK AHEAD) TRIAL

**DOI:** 10.1093/geroni/igae098.0386

**Published:** 2024-12-31

**Authors:** Charles Semelka, Rebecca Neiberg, Peter Huckfeldt, Kathleen Hayden, Haiying Chen, Lynne Wagenknecht, Mark Espeland, Denise Houston

**Affiliations:** Wake Forest University School of Medicine, Winston-Salem, North Carolina, United States; Wake Forest University School of Medicine, Winston-Salem, North Carolina, United States; University of Minnesota School of Public Health, Minneapolis, Minnesota, United States; Wake Forest University School of Medicine, Winston-Salem, North Carolina, United States; Wake Forest University School of Medicine, Winston-Salem, North Carolina, United States; Wake Forest University School of Medicine, Winston-Salem, North Carolina, United States; Wake Forest University School of Medicine, Winston-Salem, North Carolina, United States; Wake Forest University School of Medicine, Winston Salem, North Carolina, United States

## Abstract

The Look AHEAD trial intensive lifestyle intervention (ILI) was associated with better mobility in adults with type 2 diabetes and overweight/obesity. The objective of this study is to describe the post-intervention impact on disability in older adults. Disability was assessed using the Pepper Assessment Tool for Disability (PAT-D) at the first post-trial visit and three subsequent study visits during the observational phase. Disability was defined by self-reported responses ‘of a lot of difficulty’ or ‘unable to do’ on any item within the PAT-D subscales for Basic Activities of Daily Living (BADL), Instrumental Activities of Daily Living (IADL), and mobility. Regression models using generalized estimating equations were used to examine the association between intervention assignment and BMI across post-trial visits with disability prevalence, adjusting for age, sex, race/ethnicity, CVD history, and repeated measures. The mean±SD age and BMI of participants (n=1299) at the first post-trial visit was 68.7±5.6 years and 34.4±6.1 kg/m2, 61% were female, 69% were White, 8% had CVD history at baseline, and 51% were assigned to ILI. Over a median 9 years of post-trial follow-up, disability increased in BADLs from 3.1% to 12.6%, IADLs from 3.5% to 22.7%, and mobility from 21.9% to 46.7%. Participants with a higher BMI (35+ vs. <30 kg/m2) had increased odds of disability in IADLs (OR [95% CI]: 2.0 [1.5-2.8]) and mobility (2.2 [1.7-2.9]). There was no association between prior assignment to ILI and disability (p≥0.78). In conclusion, we found clinically relevant differences in late-life disability by post-trial BMI status.

